# Bone Fracture History in Women With First Episode or With Persistent Anorexia Nervosa

**DOI:** 10.1002/erv.3153

**Published:** 2024-11-21

**Authors:** Mariana P. Lopes, Sana Ahmed, Lily Beaman, Brendon Stubbs, Iain C. Campbell, Ulrike Schmidt, Lauren Robinson

**Affiliations:** ^1^ Department of Psychological Medicine Centre for Research in Eating and Weight Disorders Institute of Psychiatry, Psychology & Neuroscience King's College London London UK; ^2^ Nutrition Department School of Public Health University of São Paulo São Paulo Brazil; ^3^ Social, Genetic and Developmental Psychiatry (SGDP) Centre Institute of Psychiatry Psychology & Neuroscience King's College London London UK; ^4^ Syed Babar Ali School of Science and Engineering Lahore University of Management Sciences Lahore Pakistan; ^5^ Psychology and Neuroscience Institute of Psychiatry Kings College London London UK; ^6^ Centre for Sport Science and University Sports University of Vienna Vienna Austria; ^7^ Department of Psychological Medicine Institute of Psychiatry, Psychology and Neuroscience King's College London London UK; ^8^ Eating Disorders Outpatient Unit South London and Maudsley NHS Foundation Trust London UK

**Keywords:** amenorrhoea, anorexia nervosa, bone mineral density, early intervention, fractures

## Abstract

**Objective:**

To investigate fractures history in women with first episode anorexia nervosa (AN) (FE‐AN: ≤ 3 years duration) and those with persistent AN (P‐AN: ≥ 7 years), compared to healthy controls (HC).

**Method:**

One hundred nineteen women (FE‐AN = 49, P‐AN = 46 and HC = 24) completed online questionnaires on eating disorders symptoms, their menstrual and their fracture history.

**Results:**

Average illness duration was 1.9 years (SD = 0.8) in FE‐AN and 15.3 years (SD = 8.5) in P‐AN. Lifetime history of all fractures, including stress fractures, was higher in AN groups (FE‐AN = 33.3%; P‐AN = 37.8%) than in HC (4.2%, *p* < 0.001). P‐AN participants were 13.4 times more likely to report a fracture compared to HC, irrespective of age, whereas F‐AN participants were 10.3 times more likely. In P‐AN, higher BMI, shorter duration of amenorrhoea and history of pregnancy were inversely associated with fracture number.

**Conclusions:**

There is an increased risk of bone fracture even in the early stages of AN. This could be related to a time lapse between the initial symptoms of AN and formal diagnosis. This suggests guidelines recommending bone screening after 2‐years of persistent low weight for adults should be revisited, and the risk of bone problems should be part of the dialogue between clinicians, patients and carers at the earliest opportunity.

**Summary**

- Lifetime fracture rates were similar between females with persistent anorexia nervosa (7 years or more) and those with early‐stage anorexia nervosa (3 years or less). These were 37.8% and 33.3%, respectively, and were significantly higher than the rate in healthy controls (4.2%). Females with persistent anorexia nervosa were 13.4 times more likely and those with early‐stage anorexia nervosa were 10.3 times more likely to report fractures compared to healthy controls.
- Higher BMI, shorter duration of amenorrhoea and a history of pregnancy were associated with lower fracture numbers in participants with persistent anorexia nervosa.
- Increased fracture risk was observed even in early‐stage anorexia nervosa, highlighting the importance of early intervention and bone health monitoring. Current guidelines relying on 2 years of persistent low weight may underestimate fracture risk, necessitating a re‐evaluation to improve early detection and intervention strategies for bone health in anorexia nervosa.

## 1 Introduction and Aims

Bone loss is a serious physical consequence of anorexia nervosa (AN; Misra, Golden, and Katzman 2016; Grinspoon et al. 2000), with osteopenia and osteoporosis affecting 54% and 38% of adult women with AN, respectively (Grinspoon et al. 2000): these conditions increase the risk of bone fractures (Kanis et al. 2019; Vestergaard et al. 2003). A meta‐analysis has shown that women with AN are 84% more likely to experience a fracture than healthy women (Solmi et al. 2016). A large population‐based cohort study (> 9000 participants) found that eating disorders (EDs) increase the risk of hip fracture, osteoporotic fracture, fracture in general, and non‐fracture fall, irrespective of sex and age (Axelsson et al. 2022). The increased fracture risk is reported to persist for more than 10 years post‐AN diagnosis (Lucas et al. 1999; Frølich et al. 2020) and to persist even after recovery. Remission from AN is associated with a significant reduction in the risk of fracture incidence, decreasing from 260% higher than healthy controls (HC) during active illness, to 70% higher in the remitted state (Frølich et al. 2020). Given that the primary treatment approach for osteoporosis is weight and menstrual recovery, and that there are limited pharmacological treatment options, it is important to establish the impact of duration of illness on fracture risk in AN (National Institute for Health and Care Excellence 2017; Robinson et al. 2017).

Risk‐of‐fracture, the ratio of applied load to bone strength, increases when there is either lower bone strength and/or higher applied load (Bachmann et al. 2016). Bone strength is determined by the mineral content of the bone, bone spatial distribution and its microarchitecture (Friedman 2006; Thakker et al. 2017). Bone mineral density (BMD) is the gold‐standard test for diagnosing osteoporosis and estimating fracture risk (Kanis et al. 2019). Loss in BMD results from multifactorial processes (Lopes et al. 2022). In EDs, and in AN specifically, the main factors associated with impaired bone health are low weight, longer duration of illness and amenorrhoea (Solmi et al. 2016; Hübel et al. 2019; Lopes et al. 2022). It is unclear, however, to what extent other ED symptoms and behaviours (e.g., binge‐eating, self‐induced vomiting, laxative misuse and compensatory exercise) affect bone health (Robinson et al. 2019).

In a study of Danish women, illness duration prior to referral to treatment, age of onset, and lowest BMI independently predicted fractures (Frølich et al. 2020). As peak bone development occurs from the teenage years to the mid‐20s (i.e., across the peak period of onset of EDs (Solmi et al. 2022)), disrupted bone development may lead to weakened bones throughout adulthood (Robinson, Micali, and Misra 2017). Neurobiological, neuroimaging and behavioural evidence, mainly in AN, indicates that after an illness duration of > 3 years, achieving full recovery from the illness is more difficult (Russell et al. 2019; Lang et al. 2016; Le Grange and Loeb 2007; Treasure, Stein, and Maguire 2015). Early intervention models such as FREED (First Episode Rapid Early Intervention for Eating Disorders) use a 3‐year illness duration cut‐off to delineate early‐stage illness. Data indicate that 20%–30% of AN cases have a persistent form of illness (Dalton et al. 2020; Kotilahti et al. 2020; Solmi et al. 2024), presenting treatment and management challenges and long‐term physical and psychological complications (Walsh 2013; Frostad and Bentz 2022). It remains unclear how the risk of fractures varies across different disease duration lengths. In the present study, we have used such criteria, to assess fracture history as an indicator of bone health, in individuals who have a first episode recent onset form of AN (FE‐AN, < 3 years) versus those with persistent AN (P‐AN, > 7 years) compared to HC. We have also examined how clinical characteristics, behaviours and history of the illness are associated with fractures in the early and later stages of AN.

## 2 Methods

### 2.1 Participants and Study Design

In this cross‐sectional online study, 116 participants (FE‐AN *n* = 45, P‐AN *n* = 49 and HC *n* = 24) were recruited as part of the ‘First Episode Eating Disorders and Markers of Bone Health’ longitudinal study, which was adapted due to the COVID‐19 pandemic. From August 2020 to February 2021, females aged 18–65 years, with a primary diagnosis of AN, together with a normal weight HC group, were recruited from the South London and Maudsley NHS Foundation Trust, through online advertisements in social media in the UK, via ED services with email and lists of participants from previous studies at King's College London.

Potential participants were screened by phone using the Structured Clinical Interview for the Diagnostic and Statistical Manual of Mental Disorders (DSM‐5; First et al. 2016). For inclusion, females had to fulfil the criteria for AN according to DSM‐5 (restricting or binge‐eating/purging type) have a BMI < 18.5 kg/m^2^, and an illness duration of < 3 years (FE‐AN) or > 7 years (P‐AN), assessed by an ED trajectory questionnaire (Brown and Harris 1989; Flynn et al. 2021; Reas and Rø 2018). HC had to be free of current or past psychiatric disorders. Exclusion criteria included the presence of chronic physical disease or pregnancy. Participants provided informed consent before study participation. The study was approved by the London–Queen's Square Research Ethics Committee (REC ref: 20/LO/0211). Participants were compensated with £10 after completion of the survey.

### 2.2 Measurements

Participants completed an online set of questionnaires through the ‘Qualtrics’ Platform (Qualtrics 2021). Sociodemographic characteristics, and variables known to impact bone health, such as smoking, skin colour, and current, lowest‐ever (since illness onset) and highest‐ever BMI were also recorded.

### 2.3 Eating Disorder Symptoms and Behaviours

The Eating Disorder Examination‐Questionnaire Version 6.0 (EDE‐Q 6.0) was used to assess ED symptoms over the past 28 days (Fairburn and Beglin 2008), generating global and subscales scores (restraint, eating concern, weight concern and shape concern). The Eating Disorder Diagnostic Scale (EDDS; Stice, Telch, and Rizvi 2000) was used to classify the presence and frequency of ED behaviours (restricting, binge‐eating/purging, self‐induced vomiting, laxative misuse and compensatory exercise) over the past 3 months.

### 2.4 Menstrual Function and Bone Fracture History

A history of menstrual function and bone fractures was collected using questionnaires from the Avon Longitudinal Study of Parents and Children (ALSPAC; ALSPAC Executive Committee, n.d.). Participants reported (in years), age at menarche, age at natural menopause, history of missed menstrual periods for > 6 months (excluding pregnancy or contraceptive pills), total duration of amenorrhoea, occurrence of periods in the past 3 and 12 months, reason for absent periods in the past 3 and/or 12 months (surgery, pregnancy/breastfeeding, contraception, chemotherapy or radiation therapy, no obvious reason/menopause, periods not started yet, other reasons), date of last menstrual period, and pregnancy and hormonal contraception history. Participants reproductive lifespan was calculated according to three parameters: (1) expected length, as current age minus age at menarche; (2) reported length, as age at last period minus age at menarche; and (3) amenorrhoea‐adjusted length, reported length minus the duration of amenorrhoea (Magnus et al. 2018).

Participants were asked for any history of broken bones including body region (finger, toe, arm/shoulder, leg, spine, other), number of fractures, age at fracture, how the fracture occurred (during serious accident, high impact activity, low impact activity, sport or due to another reason). They also reported history of stress (hairline) fracture(s), body region affected (foot, leg, wrist, arm above wrist, other), and whether any stress fracture was sport‐related.

### 2.5 Physical Activity

The Simple Physical Activity Questionnaire (SIMPAQ; Rosenbaum et al. 2020) is a five‐item tool for assessing physical activity and sedentary behaviour validated for use in people with mental illness. It assesses walking time (h/week) and moderate‐vigorous physical activity time (MVPA) (h/week) over the past week. After completing the SIMPAQ, participants were asked how much their physical activity behaviour had changed due to the COVID‐19 lockdown.

### 2.6 Mood and Alcohol Consumption

The Depression Anxiety Stress Scale 21‐item version (DASS‐21; Lovibond and Lovibond 1996) was used to assess symptoms of depression, anxiety and stress (as subscales) over the past 7 days. The self‐reported version of the Alcohol Use Disorders Identification Test (AUDIT) was used to assess alcohol consumption, drinking behaviours and alcohol‐related problems (Bohn, Babor, and Kranzler 1995).

### 2.7 Statistical Analyses

Statistical analyses were conducted using SPSS 21.0 (Statistical Package for Social Science Inc., Chicago, Illinois USA) software. Variables are presented as means (standard deviation) with minimum and maximum values, 95% confidence interval or frequencies (%), or medians, adopting a significance level of 5%. The normality of continuous variables was assessed using the Kolmogorov–Smirnov test, and homogeneity was assessed using the Levene test. Non‐normally distributed variables were log‐transformed.

Differences between the groups (FE‐AN, P‐AN and HC) were investigated using independent *t*‐tests and ANOVAs for continuous variables, and Chi‐squared tests for categorical variables. Logistic regressions were performed to determine the risk of fractures (odds ratio) in the three groups. Multiple linear regression models were used to assess the relationship between fractures (independent variable) and ED history (dependent variables), while including age, EDE‐Q global score and AN subtype as covariates in all analyses for both the P‐AN and FE‐AN groups. A sensitivity analysis was conducted including only premenopausal females to assess whether the inclusion of postmenopausal women would have impacted the results.

## 3 Results

### 3.1 Demographic and Clinical Characteristics

Demographic and clinical characteristics are shown in Table 1. The FE‐AN group was significantly younger than the HC and P‐AN groups. The P‐AN group had a lower age of AN onset, and a longer illness duration than the FE‐AN group (FE‐AN: median = 1.9 years 95% CI 1.67–2.09 and P‐AN: median = 12.9 years, 95% CI 11.2–15.8, *p* < 0.001). Proportions of those classified as restricting or binge‐purge type AN differed significantly between groups: In the FE‐AN group, 36.7% were restricters and 63.3% binge‐purgers. This compares to 73.3% and 26.7%, respectively in the P‐AN group. As expected levels of behaviours defining AN binge‐purge sub‐types, such as binge eating (BE) episodes, laxative use and self‐induced vomiting were aligned with sub‐type diagnosis. In the P‐AN group only, those with AN binge‐eating/purging type had longer illness duration and higher EDE‐Q weight concern scores. The P‐AN group had a lower current and lower ‘lowest‐ever’ BMI than the FE‐AN group. Both AN groups reported lower current BMI and lowest‐ever BMI than the HC. Highest‐ever BMI was significantly lower in the AN groups than HC but did not differ significantly between the FE‐AN and P‐AN groups. As Supporting Information S1: Table A1 shows, no significant differences were found between the three groups in terms of sociodemographic variables, smoking and alcohol intake. The FE‐AN and P‐AN groups had significantly higher DASS total scores and also stress, anxiety and depression subscale scores than the HC, however, these scores did not differ significantly between the two AN groups.

**TABLE 1:** Demographic, anthropometric and clinical characteristics of female adults with first episode (FE) or severe and enduring (SE) anorexia nervosa (AN), and of healthy controls.

Sample characteristics	FE‐AN (*n* = 49)		P‐AN (*n* = 45)		HC (*n* = 24)	
	Mean (SD), median or frequencies (*n*)	95% CI	Mean (SD), median or frequencies (*n*)	95% CI	Mean (SD), median or frequencies (*n*)	95% CI
Age (year)	24.2 (5.9)^a,^*^,b,^**	22.6 to 25.9	29.0 (8.6)	26.5 to 31.6	32.7 (14.6)	26.5 to 38.8
AN restricting type	36.7% (*n* = 18)^b,^***		73.3% (*n* = 33)			
AN binge‐eating/purging type	63.3% (*n* = 31)^b,^***		26.7% (*n* = 12)			
Duration of illness (year)	1.9 (0.8)^b,^***	1.7 to 2.1	15.3 (8.5)	12.7 to 17.8		
AN onset age (year)	21.7 (5.9)^b,^***	19.9 to 23.4	16.7 (5.4)	14.8 to 18.5		
Current BMI (kg/m^2^)	17.9 (2.5)^a,^***^,b,^**	17.1 to 18.7	15.9 (1.9)^a,^***	15.3 to 16.5	23.6 (4.1)	21.9 to 25.3
Lowest‐ever BMI (kg/m^2^)	16.1 (2.3)^a,^***^,b,^***	15.3 to 16.8	13.9 (1.6)^a,^***	13.4–14.4	21.4 (3.5)	19.9 to 22.8
Highest‐ever BMI (kg/m^2^)	21.4 (3.4)^a,^**	20.4 to 22.5	19.8 (2.7)^a,^***	18.9 to 20.6	25.2 (4.8)	23.2 to 27.2
AUDIT total score	5.8 (6.9)	3.8 to 7.9	3.4 (5.1)	1.8–5.0	3.2 (3.3)	1.7 to 4.6
DASS total score	29.4 (15.1)^a,^***	24.8 to 34.0	30.3 (12.0)^a,^***	26.5 to 34.1	6.7 (7.2)	3.6 to 9.8
Menstrual health						
Age of menarche (year)	13.1 (2.0)	12.5 to 13.7	13.5 (2.8)	12.0 to 14.3	12.3 (1.5)	11.7 to 12.9
History of amenorrhoea	39.6% (*n* = 19)^b,^*		66.7% (*n* = 30)^a,^**		16.7% (*n* = 4)	
Current amenorrhoea	25.5% (*n* = 12)		46.7% (*n* = 21)^a,^*		16.7% (*n* = 4)	
Duration of amenorrhoea (year)	1.5 (1.6)^b,^***	1.0–2.0	5.0 (3.0)^a,^***	4.1 to 6.0	0.7 (1.4)	0.0 to 1.3
History of hormone contraception	45.8% (*n* = 22)		50.0% (*n* = 22)		62.5% (*n* = 15)	
Current OCP	83.3% (*n* = 40)		81.8% (*n* = 36)		84.2% (*n* = 16)	
History of pregnancy	8.2% (*n* = 4)		17.8% (*n* = 8)		17.4% (*n* = 4)	
ED symptoms and behaviours						
EDE‐Q global score	3.5 (1.0)^a,^***	3.2–3.8	3.7 (1.3)^a,^***	3.2 to 4.1	0.7 (1.0)	0.3 to 1.2
Restricting behaviour	71.4% (*n* = 35)^b,^*		44.4% (*n* = 20)		8.3% (*n* = 2)	
Monthly average/past 3 months	6.8 (6.3)^a,^***^,b,^*	5.0–8.6	4.1 (6.2)^a,^*	2.1 to 6.0	0.8 (3.3)	
Binge eating episode	64.6% (*n* = 31)^b,^***		22.2% (*n* = 10)			
Monthly average/past 3 months	6.1 (9.3)^a,^***^,b,^***	3.4 to 8.9	4.1 (15.2)	−0.5 to 8.6		
Self‐induced vomiting	49.0% (*n* = 24)^b,^*		26.7% (*n* = 12)			
Monthly average/past 3 months	6.2 (11.4)^a,^***^,b,^*	2.9 to 9.5	6.4 (20.3)	0.2–12.7		
Laxative/diuretics use	30.6% (*n* = 15)		24.4% (*n* = 11)		4.2% (*n* = 1)	
Monthly average/past 3 months	2.2 (4.5)^a,^*	0.9 to 3.5	1.9 (4.9)	0.4 to 3.4	0.0 (0.2)	0.0 to 0.1
Compensatory exercise	79.6% (*n* = 39)^b,^**		46.7% (*n* = 21)		4.2% (*n* = 1)	
Monthly average/past 3 months	7.1 (6.1)^a,^***^,b,^*	5.3 to 8.8	3.9 (5.9)^a,^*	2.1–5.7	0.4 (2.0)	−0.4 to 1.3
Physical activity						
Walking (h/week)	0.7 (0.7)^b,^*	0.5 to 0.9	1.3 (1.3)^a,^**	0.9 to 1.7	0.4 (0.6)	0.2 to 0.7
MPVA (h/week)	8.1 (8.3)	5.6 to 10.5	13.0 (13.7)^a,^**	8.9 to 17.2	4.7 (5.9)	2.2 to 7.2

*Note:* ANOVA post‐hoc Bonferroni and Chi squared tests, *p < 0.05, **p < 0.01, ***p < 0.001 versus ^a^HC and ^b^P‐AN.

Abbreviations: AN: anorexia nervosa; AUDIT: Alcohol Use Disorders Identification Test; BMI: body mass index; CI: confidence interval; DASS: Depression, Anxiety and Stress Scale; EDE‐Q: Eating Disorder Examination‐Questionnaire; FE: first episode (≤ 3 years); HC: healthy controls; MPVA: moderate and vigorous physical activity; *n*: sample size; OCP: oral contraceptive pill; P: persistent (≥ 7 years); *p: p‐*value; SD: standard deviation.

Regarding menstrual functioning (Table 1), the P‐AN group reported the highest rates of current (46.7%) and historic (66.7%) amenorrhoea, followed by the FE‐AN group (25.5% and 39.6%, respectively), and (16.7% and 16.7%, respectively) of HC participants. These differences were significant for both current and historic amenorrhoea in the P‐AN versus HC, and for historic amenorrhoea in the FE‐AN compared to the P‐AN group but not to the HC. The median duration of amenorrhoea in the P‐AN group was 4.6 years (96% CI 3.6–8.0), that is, longer than in the FE‐AN group (0.92 years [95% CI 0.83–1.42]) and the HC (0.29 years [95% CI 0.0–0.6]). Age of menarche, history and current use of oral contraceptive pill (OCP), and pregnancy history were similar between all groups. The reported reproductive lifespan was not different between P‐AN and HC, but was significantly lower in the FE‐AN group (FE‐AN: *M* = 10.1 years, SD = 6.2; P‐AN: *M* = 15.5 years, SD = 8.5; HC: *M* = 19.2 years, SD = 11.8). However, the P‐AN group achieved only 78.0% of the expected lifespan (*p* = 0.006), while the FE‐AN group achieved 85.4% (not statistically different), compared to HC (97.0%). The level of achievement for amenorrhoea‐adjusted lifespan was significantly lower in the P‐AN group (63.3%) and the FE‐AN group (82.4%), compared to both the HC group (96.5%). The duration of hormonal contraception was higher in the HC group (*M* = 4.2 years, SD = 7.2) compared to the AN groups (FE‐AN: *M* = 0.5 years, SD = 1.1, *p* < 0.0001; P‐AN: *M* = 1.3 years, SD = 2.9, *p* = 0.008), for more details see Supporting Information S1: Table A1. Only six participants (P‐AN = 1, HC = 5) were older than 50 years and postmenopausal, all of whom had reached menopause at or after the age of 48.

EDE‐Q total scores and subscales were similar between the P‐AN and FE‐AN groups, and as expected significantly higher in both AN groups than in the HC (Table 1). In the HC group, four participants reported engaging in ED behaviours, specifically in restricting behaviour (*n* = 2), laxative use (*n* = 1), and compensatory exercise (*n* = 1). Participants in the FE‐AN reported a significantly higher number of times of compensatory exercise than those with P‐AN. Both FE‐AN and P‐AN reported more weekly walking hours than HC, and P‐AN only also reported a significantly higher number of hours of MVPA per week than the HC group. The majority of both the FE‐AN (52.1%) and HC (47.6%) groups reported an increase in physical activity due to the COVID‐19 lockdown. In contrast, 59.1% of the P‐AN group maintained similar levels, with 29.5% reporting an increase. Reductions in physical activity were reported by 8.3% of the FE‐AN group, 11.4% of the P‐AN group and 19% of the HC group. However, no statistical difference was observed between the three groups.

Vitamin D and calcium supplementation were more prevalent in the AN groups than in the HC group (FE‐AN = 20.8%, *n* = 10, P‐AN = 46.7%, *n* = 21, and HC = 16.7%, *n* = 4, Chi^2^ = 9.9; *p* = 0.007), and for calcium (FE‐AN = 6.3%, *n* = 3, P‐AN = 31.1%, *n* = 14, and HC = 0.0%, *n* = 0, Chi^2^ = 16.7; *p* < 0.001).

### 3.2 Fracture History

A fracture history was most common in the P‐AN, followed by FE‐AN and HC (Table 2). Just over a third (39.3%) of the total AN sample reported a lifetime history of fracture (33.3% in FE‐AN and 37.8% in P‐AN). The rates of reported all lifetime fractures (including stress fractures) in both AN groups were significantly higher than the 4.2% in the HC (both *p* = 0.001). FE‐AN participants reported a significantly higher lifetime rate of stress fractures (20.8%) than P‐AN (8.9%), whereas HC participants reported no such fractures. A logistic regression adjusted for age showed the odds ratios for reporting a lifetime history of all fractures in both the AN groups were higher than in the HC (FE‐AN: OR = 10.3, 95% CI 1.2–85.8, *p* = 0.031 and P‐AN: OR = 13.45, 95% CI 1.7–109.2, *p* = 0.015). After the AN onset, the history of fractures (FE‐AN: 10.2% and P‐AN: 22.2%) and the history of stress fractures (FE‐AN: 6.1% and P‐AN: 6.7%) did not significantly differ between the groups. The P‐AN group reported a higher number of post‐onset fractures (P‐AN: *M* = 0.6, Min = 0, Max = 5 vs. FE‐AN: *M* = 0.2, Min = 0, Max = 2; *p* = 0.046), but a similar number of stress fractures, when compared to the FE‐AN group (FE‐AN: *M* = 0.4, Min = 0, Max = 1 vs. P‐AN: *M* = 2.0, Min = 1, Max = 3; *p* = 0.094). In contrast, the FE‐AN group reported a higher number of post‐onset stress fractures than the P‐AN group. No significant difference was observed in lifetime fracture history between AN subtypes in either FE‐AN (restricting: 38.9% [7/18] and binge‐eating/purging = 32.3% [10/31]) or P‐AN (restricting: 33.3% [11/33] and binge‐eating/purging = 50.0% [6/12]). The median number of lifetime fractures did not significantly vary according to subgroups (FE‐AN restricting: median = 0.0 (Lang et al. 2016), FE‐AN binge‐eating/purging: median = 0.5 (Robinson et al. 2017), P‐AN restricting: median = 0.0 (Russell et al. 2019), P‐AN binge‐eating/purging: median = 0.5 (Robinson et al. 2017)).

**TABLE 2:** Fractures in female adults with first episode (FE‐AN) or persistent (P‐AN) anorexia nervosa (AN), and in healthy controls.

Reported fractures	FE‐AN (*n* = 49)	P‐AN (*n* = 45)	HC (*n* = 24)
	Mean [min–max] or frequencies (*n*)	Mean [min–max] or frequencies (*n*)	Mean [min–max] or frequencies (*n*)
All fractures, including stress fractures			
Lifetime history	33.3% (*n* = 16)^b,^**	37.8% (*n* = 17)^b,^**	4.2% (*n* = 1)
Lifetime number	1.3 [0–20]	1.8 [0–19]^b,^*	0.1 [0–2]
Before AN onset history	26.5% (*n* = 13)	24.4% (*n* = 11)	
Before AN onset number	2.2 [0–7]	0.5 [0–2]	
After AN onset history	12.2% (*n* = 6)	22.2% (*n* = 10)	
After AN onset number	2.0 [1–3]^c,^**	7.0 [6–8]	
Fractures			
Lifetime history	29.2% (*n* = 14)^b,^*	37.8% (*n* = 17)^b,^**	4.2% (*n* = 1)
Lifetime number	1.0 [0–17]^b,^*	1.7 [0–18]^b,^*	0.1 [0–2]
Before AN onset history	22.4% (*n* = 11)	24.4% (*n* = 11)	
Before AN onset number	0.8 [0–17]	0.4 [0–4]	
After AN onset history	10.2% (*n* = 5)	22.2% (*n* = 10)	
After AN onset number	0.2 [0–2]^c,^*	0.6 [0–5]	
Stress fractures			
Lifetime history	20.8% (*n* = 10)^b,^**^,c,^*	8.9% (*n* = 4)^b,^**	0% (*n* = 0)
Lifetime number	0.3 [0–3]^b,^*	0.2 [0–3]	
Before AN onset history	12.2% (*n* = 6)	2.2% (*n* = 1)	
Before AN onset number	0.8 [0–3]^c,^**	0.1 [0–1]	
After AN onset history	6.1% (*n* = 3)	6.7% (*n* = 3)	
After AN onset number	0.4 [0–1]	2.0 [1–3]	
Fracture history^a^			*Reference*
Wald	4.9	5.9	5.9
Odds ratio	10.3	13.45	
95% CI	1.2–85.8	1.7–109.2	
*p*	**0.031**	**0.015**	0.051

*Note:* Comparisons between groups by Independent *t*‐Test and Chi‐squared tests after Fisher's Exact Test, *p < 0.05, **p < 0.01, ***p < 0.001 versus ^b^HC and ^c^P‐AN.

Abbreviations: AN: anorexia nervosa; CI: confidence interval; FE: first episode (≤ 3 years); HC: healthy controls; *n*: sample size; P: persistent (≥ 7 years); *p: p*‐value.

^a^ Logistic regression for fracture history in FE‐AN and P‐AN, and healthy controls groups, adjusted for age. Values in bold denote statistical significance (*p* < 0.05).

**FIGURE 1:**
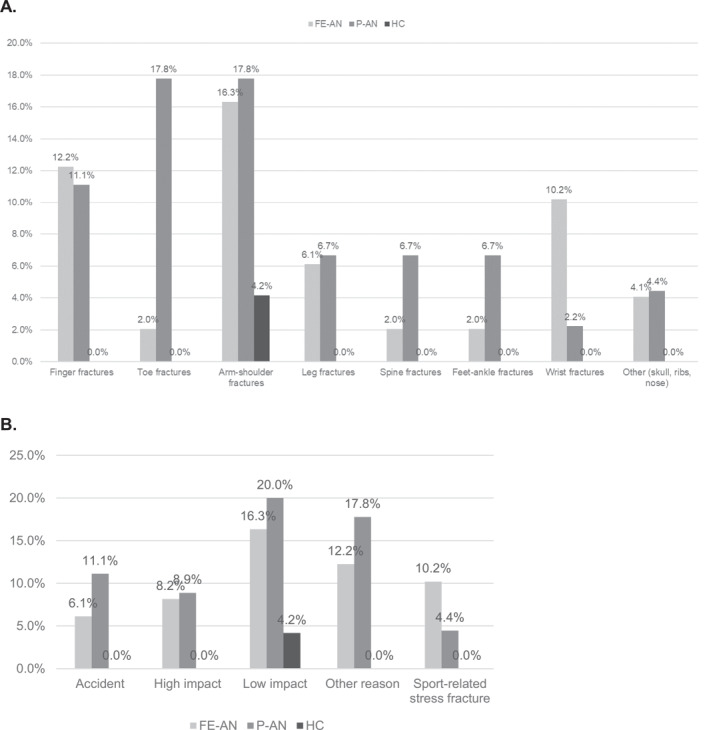
Fracture characteristics reported by participants with first episode (FE) or persistent anorexia nervosa (P‐AN), and healthy controls. Figure shows the accumulative sum of fractures reported by people with first episode (FE‐AN) or persistent anorexia nervosa (P‐AN), and in healthy controls, according to panel (A) body region affected and (B) reason for fractures, other reasons reported included physical fights and lifting objects.

Figure 1 shows body regions and reasons for fractures reported by participants. Visual analysis indicates that in FE‐AN, the most affected regions were fingers, arms and feet, while in P‐AN, toes, spine and arms/shoulders were most affected. The main reasons reported for fractures in FE‐AN were low and high‐impact activities, with sports activities also identified as reasons for stress fractures. The primary reasons for fractures in P‐AN were high‐impact activities and accidents. Participants also described other reasons for fractures (e.g., physical fights and lifting objects) and the reports included fractures as a consequence of ‘fainting and falling’ and ‘lifting a suitcase’.

### 3.3 ED History/Symptom Associations With Fractures

**FIGURE 2:**
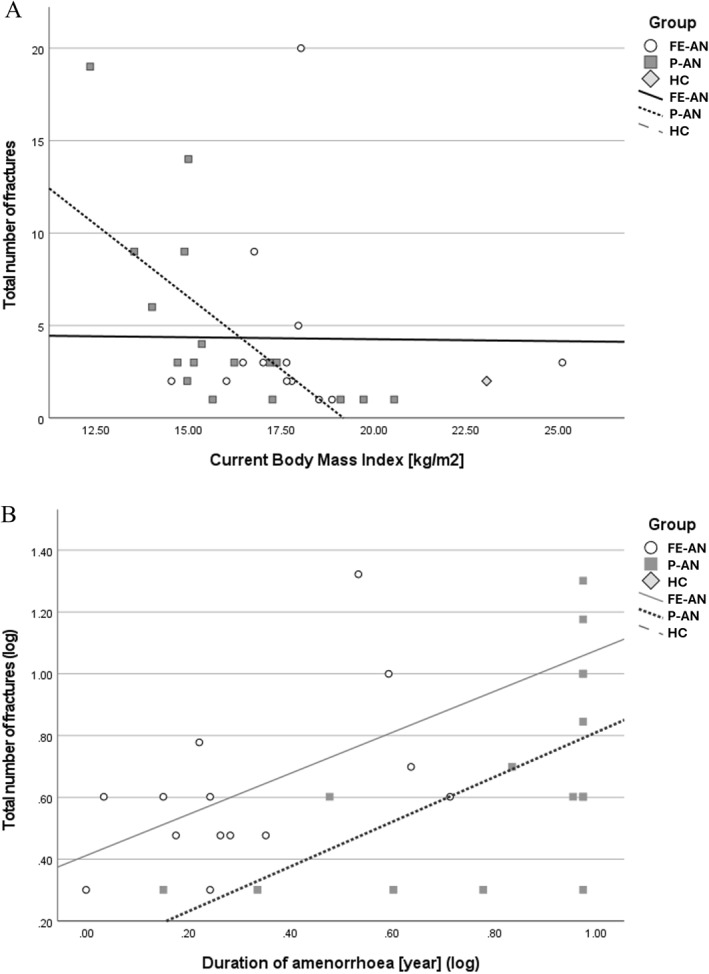
Association of body mass index (BMI) and duration of amenorrhoea with number of fractures in people with first episode (FE‐AN) or persistent anorexia nervosa (P‐AN), and in healthy controls. Association of number of reported fractures in linear regression models adjusted for age for panel (A). Body mass index (FE‐AN: *β* = −0.001, 95% CI −0.374 to 0.371, *p* = 0.994; P‐AN: *β* = −0.320, 95% CI −1.237 to −0.061, *p* = 0.031) and (B) duration of amenorrhoea (FE‐AN: *β* = 0.118, 95% CI −0.229 to 0.634, *p* = 0.472; P‐AN: *β* = 0.384, 95% CI 0.180 to 0.937, *p* = 0.013).

Linear regression analyses were conducted to examine the associations between the number of fractures and ED history/symptoms (illness duration and amenorrhoea duration), in both FE‐AN and P‐AN groups (Supporting Information S1: Table A2). In the P‐AN group, history of pregnancy (*β* = −0.363, 95% CI −7.029 to −0.306, *p* = 0.033) and higher current BMI (*β* = −0.320, 95% CI −1.237 to −0.061, *p* = 0.031) were associated with lower number of fractures, whilst longer duration of amenorrhoea (*β* = 0.384, 95% CI 0.180–0.937, *p* = 0.013) was associated with higher number of fractures (irrespective of age, EDE‐Q score and AN subtype) (Figure 2). No significant associations were found between the number of fractures and ED history in participants with FE‐AN.

After conducting a sensitivity analysis including only premenopausal participants, the previously significant age difference between the FE‐AN and HC groups was no longer observed, and the association between BMI and the total number of fractures in the P‐AN group was no longer significant. The other results remained consistent with the whole sample analysis. Supporting Information S1: Tables A3–A5 provide detailed information on demographics, clinical characteristics, fracture history and linear regression results for this subgroup.

## 4 Discussion

We examined bone fracture history in relation to illness stage in a cohort of adult women with persistent AN (P‐AN) or early‐stage AN (FE‐AN). The groups differed significantly in relation to the proportions with different AN subtypes, that is, with one‐third of the FE‐AN group and two‐thirds of the P‐AN group having the restricting subtype. As expected, the P‐AN group was slightly older, had a longer average illness duration (by 13.4 years) and given the high proportion of restrictors, also had a lower BMI (by 2 kg/m^2^). They had 21% more cases of current amenorrhoea, which, on average, lasted 3.5 years longer, than in those with FE‐AN. Lifetime fracture history was, however, similar in both AN groups and significantly different from the HC (P‐AN: 37.8%, FE‐AN: 33.3%, HC: 4.2%). The likelihood of a history of bone fracture was 13.4 times higher in the P‐AN group and 10.3 times higher in the FE‐AN group than in the HC, irrespective of age. The P‐AN group reported more fractures after AN onset than the FE‐AN group (probably due to their higher age and longer illness duration). The P‐AN group reported fewer stress fractures prior to the onset of AN than the FE‐AN group. In the P‐AN group, the main areas of fractures were spine/toes arising from high‐impact/accidents, and in FE‐AN, they were in fingers/feet due to low‐impact/high‐impact activities. In the P‐AN group, higher BMI, a shorter duration of amenorrhoea and a history of pregnancy were inversely associated with fracture number. These data highlight the importance of preventing bone loss in AN, including in people in the early stages of the illness.

Our findings accord with studies reporting higher fracture rates and/or fracture risk in people with AN (i.e., than HC) (Axelsson et al. 2022; Lucas et al. 1999; Frølich et al. 2020; Thakker et al. 2017; Nagata et al. 2017; Faje et al. 2014). The FE‐AN group also reported a higher number of stress fractures prior to the onset of AN than the P‐AN group possibly because of a time delay between symptom onset and a formal diagnosis being made (Faje et al. 2014). In the P‐AN group, fractures were most frequent in the spine, toes and arm/shoulder and in the FE‐AN group, fractures were most common in fingers, arms, feet and wrist. Fractures of the spine, hip and forearm have been reported to be late consequences that may occur more than 24 years after the AN diagnosis (Lucas et al. 1999; Frølich et al. 2020; Nagata et al. 2017) but, in fact, females with AN are reported to be at increased risk of fractures across all ages and across multiple anatomical sites (Nagata et al. 2017). Participants with P‐AN cited high‐impact activities and accidents while those with FE‐AN reported low‐impact, high‐impact and sports activities, as the main causes of fractures. Fractures due to fainting followed by falling, and the lifting of objects were also reported. Our small sample size means that our findings related to regions and reasons for fractures are descriptive.

Low weight (and BMI) increases the risk of osteoporosis and fractures in younger women (Bachmann et al. 2016). In our P‐AN group, fractures were associated with current BMI, but in the FE‐AN group, no significant associations were found between fractures and ED history. In our P‐AN group, the effect of low weight on reproductive health, (i.e., inhibition of the hypothalamic‐pituitary–gonadal axis [HPG] axis) (Misra and Klibanski 2016), was associated with a history of fractures (i.e., duration of amenorrhoea and pregnancy). In the P‐AN group only, fractures were positively associated with duration of amenorrhoea (mean duration 5 years), and although not significant, a similar linear regression involving the FE‐AN group showed a similar pattern. In AN, this relationship between low oestrogen levels, amenorrhoea and low BMD is well‐established (Solmi et al. 2016; Lopes et al. 2022; Robinson, Micali, and Misra 2017b). In the P‐AN group, a history of pregnancy (reported by 17.8%) was inversely associated with fractures. As those who reported a history of pregnancies were older, with only one participant reporting pregnancy before AN onset, further investigation is necessary to establish whether this association indicates a period of recovery where a healthy weight was achieved or a later onset of AN that might have preserved the attainment of peak bone mass during adolescence. The longer duration of hormonal contraception use by HC participants, combined with the fact that withdrawal bleeds can mimic regular menstrual cycles, could have obscured actual differences in reproductive lifespan. Adding detailed chronological data to the study design on the timing of missed periods, hormonal contraception use and pregnancies will enable better comparability of the effect of the ED on reproductive health. In women in general, approximately two‐thirds of the fracture risk can be predicted from premenopausal BMD, which can be negatively affected, for example, by AN symptoms in adolescence such as secondary amenorrhoea and excessive exercise (Sowers 2000). Early menopause (occurring before 47) has also been associated with increased risk of osteoporosis, increased fragility fractures and mortality than later menopause (occurring after age 47 or later) (Svejme et al. 2012), and should be investigated in people with P‐AN.

### 4.1 Bone Health Care in Early and Severe Stages of AN

The finding that 30%–40% of people with P‐AN and FE‐AN reported a fracture, emphasises the need to integrate bone health care at all stages of AN. An increased risk of fractures as early as 1 year following anAN diagnosis has been reported (Vestergaard et al. 2002), but the extent to which people with AN may have an elevated risk of fractures even prior to receiving the diagnosis is uncertain (Faje et al. 2014; Vestergaard et al. 2002). A cross‐sectional study of 310 adolescent females with AN (mean illness duration, two years) found a higher incidence of prior fractures in the AN group than in HC, despite no significant reductions in BMD (Faje et al. 2014). Those with AN not only reported a peak in fracture incidence after AN onset but also had a higher lifetime prevalence of prior fractures than HC (31.0% vs. 9.4%) (Faje et al. 2014). This suggests that there could be other factors contributing to reduced bone strength, such as changes in microarchitecture, that are not assessed in regular bone scans. As peak bone development occurs during the teenage years and up to the mid‐20s, the onset of an ED, may prevent people from reaching normal peak bone mass, leaving them with weakened bones which are prone to breaking throughout adulthood (Robinson, Micali, and Misra 2017). Combining BMD with a trabecular bone score (TBS), a measurement related to bone microarchitecture parameters that can be extracted from DXA images, could be a promising tool for the prediction of fracture risk in women (Hans et al. 2011; Goel et al. 2024). Our findings emphasise the importance of early diagnosis together with early assessments of bone health.

During the early stage of AN (≤ 3 years), the disorder is relatively malleable, and symptoms may vary, potentially leading to a change in diagnosis (Treasure, Stein, and Maguire 2015). Cross‐over from AN to bulimia nervosa (BN) is common in both AN types (20%–40% for the AN restrictive type and, 50% for the binge‐eating/purging type) (Treasure, Stein, and Maguire 2015; Monteleone et al. 2011). Transition from AN (restrictive type) to BN typically occurs between 1 and 3 years of illness onset (average, 2 years) (Robinson, Micali, and Misra 2017). Therefore, in the FE‐AN group, who had had AN for an average of 2 years and where 63% were classified with the binge‐eating/purging type, it is likely that symptoms will change as the illness progresses and some of them will cross over to a BN diagnosis.

The risk of osteoporosis and fractures increases throughout the course of the ED. Lucas et al. found a cumulative incidence of any fracture, reaching 57%, 40 years after a diagnosis of AN (Lucas et al. 1999). A population‐based retrospective study tracking 9239 females and males with AN for an average of 3.4 years, found that 50% of individuals experienced their first fracture between 5.0 and 22.4 years (median = 12.2 years) after receiving a formal diagnosis of AN (Nagata et al. 2017).

In the retrospective Danish study of participants with AN, age of AN onset, nadir BMI and duration of illness before treatment, were identified as risk factors for fracture (after being followed for a median of 19.3 years) (Frølich et al. 2020). Therefore, regular assessments of BMD in people with AN are important. NICE guidelines recommend bone densitometry scans for adults with ongoing and persistent underweight after 2 years or when experiencing fractures or severe pain (National Institute for Health and Care Excellence 2017). However, our finding that the FE‐AN group, with a mean illness duration of 1.9 years (95% CI 1.7–2.1), reported higher fracture rates than HC suggests a 2‐year mark may not fully identify those in need of bone screening. It is important, therefore, for clinicians to be aware that fracture risk is high even during the early stages of AN. Accordingly, clinicians should monitor ED history/symptoms that are risk factors for osteoporosis and fractures (i.e., BMI and reproductive health as shown in this study) and symptoms (pain and fractures) in people with AN, as this may help identify those who may require referral for bone scans and mitigate the long‐term risks of osteoporosis and fracture. Awareness of and investigation of the problem will also encourage clinicians to educate patients, families and carers about the malleable physical changes that occur during the early illness stages (Frølich et al. 2020).

This might be partially explained by the fact that people with ED tend to overestimate physical activity (Keyes et al. 2015), that is they tend to have a high drive to exercise but may, not engage in much activity because of illness‐related low energy levels. The issue of bone health and physical activity is also somewhat complicated by the fact that while high‐intensity exercise is associated with increases in BMD (Le Grange and Loeb 2007), exercise is not recommended for the purpose of increasing bone mass in people with AN when they are at a very low weight (Axelsson et al. 2022; Lopes et al. 2022; Kistler‐Fischbacher, Weeks, and Beck 2021; Fazeli and Klibanski 2018). Questions associated with fracture risk and exercise/exercise patterns are likely to be resolved when, longitudinal studies employing direct measures of physical activity, for example via remote measurement/passive sensing, become more widespread (Kuehne et al. 2024).

Our data do not support an association between depressive disorders and increased fracture incidence although this has been reported by others (Pan et al. 2018). This could be related to evidence showing that depression was associated with fractures in older (> 65 years), but not younger (≤ 65 years), women (Williams et al. 2014). We did not identify any added risk (compared to HC) for lifestyle factors for example, cigarette smoking and alcohol consumption (known risk factors for osteoporosis) (Sowers 2000). This contrasts somewhat with a study, which showed that alcohol intake increased the likelihood of fracture in women between 35 and 59 years but only when combined with substantial thinness (Hemenway et al. 1988) that is, there may be multiple factors involved in this relationship.

The strength of this study is that it reveals differences in fracture history in individuals with AN with varying durations of illness and the increased risk of fracture history even in the early stages of illness. The study has some limitations. The cross‐sectional design does not allow causality to be established. The COVID‐19 pandemic prevented the collection of biochemical, anthropometrical and body composition data, that is, we relied on online and self‐reported questionnaires, and future studies should combine retrospective questionnaires with direct measurements. To address the inclusion of both premenopausal and postmenopausal females, a sensitivity analysis was conducted including only premenopausal females. Another limitation is that we did not collect data on medications known to affect bone health, such as cortisone and antacid formulations (Lespessailles and Toumi 2022). Lastly, since participants were self‐selected and may have enroled due to a history of fractures, the sample included in the present study may not be representative of all people with AN.

The study raises some questions that remain to be addressed:

1. What should be the criteria for bone scan referral in individuals in the early stages of AN? Our study and previous meta‐analysis (Lopes et al. 2022) suggest it should include a combination of symptoms and illness history that is low BMI, low fat mass, duration of amenorrhoea and history of fractures.
2. How can technology and statistical modelling be used to better predict fracture risk in people with AN? Longitudinal studies with larger sample sizes will inform how risk evolves over time and the importance of each predictor at different clinical stages of illness. These could be used in the development of fracture risk tools for example FRAX (Kanis et al. 2019), for individuals with EDs.
3. To what extent do ED symptoms and illness history predict the differential susceptibility to bone loss among individuals with AN? This is a complex issue that is increased understanding of the interplay between the onset of illness, peak bone mass, exercise patterns and recovery trajectories will be required.
4. How can we measure the impact of fractures at both individual and health system levels? Data on bone scans and fractures in people with current or past EDs will be necessary to provide a comprehensive public health assessment of short‐term, medium‐term and long‐term outcomes to inform decision‐making and better allocation of resources.

To conclude, we report bone fracture history and associated data in women at different stages of AN. Both P‐AN and FE‐AN groups reported significantly higher numbers of fractures than HC, irrespective of age. The increased fracture risk in the FE‐AN group was somewhat unexpected: it may be related to a rapid effect of the illness on bone health, and/or may also be related to a time delay between the initial symptoms of AN and the formal diagnosis. In terms of bone screening, guidelines recommending the use of 2 years of persistent low weight in adults as an indicative marker may not be sufficient for identifying those in need of investigation. Clinicians should be aware of the increased fracture risk even in the early stages of AN. A case can be made for having the risk of long‐term bone problems as an important part of the dialogue between clinicians, patients, and their carers, for example, because of the potentially motivating effects of maintaining long‐term bone health. Future studies are needed to inform criteria for bone scan referral and monitoring, to predict fracture risk in AN, to clarify the role of ED symptoms and illness history on bone loss, and to assess the public health impact of bone health in people with AN.

## Ethics Statement

The study was approved by the London–Queen's Square Research Ethics Committee (REC ref: 20/LO/0211).

## Consent

The authors have nothing to report.

## Permission to Reproduce Material From Other Sources

Not applicable.

## Supporting information

Supplementary Material

## Data Availability

The datasets used and/or analysed during the current study are available from the corresponding author on reasonable request.
