# Supplementary material for: Bone Fracture History in Women With First Episode or With Persistent Anorexia Nervosa
Source: Eur Eat Disord Rev. 2024 Nov 21;33(3):447–59. doi: 10.1002/erv.3153 (PMC11965545; doi:10.1002/erv.3153)
Supplement: Supplementary file 1 — Supplementary Material [file ERV-33-447-s001.docx]

**Appendix 1.** Additional demographic, anthropometric, and clinical characteristics of female adults with first episode (FE) or persistent anorexia nervosa (P-AN), and of healthy controls.

| **Sample characteristics** | **FE-AN (n = 49)** | | **P-AN (n = 45)** | | **HC (n = 24)** | | |
| --- | --- | --- | --- | --- | --- | --- | --- |
|  | **Mean (SD) or frequencies (n)** | **95% CI** | **Mean (SD) or frequencies (n)** | **95% CI** | **Mean (SD) or frequencies (n)** | **95% CI** |  |
| Age [year] | 24.2 (5.9)^a*, b**^ | 22.6-25.9 | 29.0 (8.6) | 26.5-31.6 | 32.7 (14.6) | 26.5-38.8 |  |
| AN Restricting type | 36.7% (n = 18)^b***^ |  | 73.3% (n = 33) |  |  |  |  |
| AN Binge-eating/purging type | 63.3% (n = 31)^b***^ |  | 26.7% (n = 12) |  |  |  |  |
| Duration of illness [year] | 1.9 (0.8)^b***^ | 1.7-2.1 | 15.3 (8.5) | 12.7-17.8 |  |  |  |
| AN onset age [year] | 21.7 (5.9)^b***^ | 19.9-23.4 | 16.7 (5.4) | 14.8-18.5 |  |  |  |
| **BMI [kg/m^2^]** |  |  |  |  |  |  |  |
| Current BMI [kg/m^2^] | 17.9 (2.5)^a***, b**^ | 17.1-18.7 | 15.9 (1.9)^a***^ | 15.3-16.5 | 23.6 (4.1) | 21.9-25.3 |  |
| Lowest-ever BMI [kg/m^2^] | 16.1 (2.3)^a***, b***^ | 15.3-16.8 | 13.9 (1.6)^a***^ | 13.4-14.4 | 21.4 (3.5) | 19.9-22.8 |  |
| Highest-ever BMI [kg/m^2^] | 21.4 (3.4)^a**^ | 20.4-22.5 | 19.8 (2.7)^a***^ | 18.9-20.6 | 25.2 (4.8) | 23.2-27.2 |  |
| **Skin colour** |  |  |  |  |  |  |  |
| Fair or very fair | 59.2% (n = 29) |  | 77.8% (n = 35) |  | 58.3% (n = 14) |  |  |
| Olive | 14.3% (n = 7) |  | 13.3% (n = 6) |  | 25.0% (n = 6) |  |  |
| Brown or dark | 26.5% (n = 13) |  | 8.9% (n = 4) |  | 16.7% (n = 4) |  |  |
| **Occupation** |  |  |  |  |  |  |  |
| Working | 44.7% (n = 21) |  | 48.8% (n = 21) |  | 45.8% (n = 11) |  |  |
| Unemployed or on unpaid leave | 12.8% (n = 6) |  | 27.9% (n = 12) |  | 16.7% (n = 4) |  |  |
| Student | 42.6% (n = 20) |  | 23.3% (n = 10) |  | 37.5% (n = 9) |  |  |
| **Household** |  |  |  |  |  |  |  |
| Living with family | 55.3% (n = 26) |  | 54.5% (n = 24) |  | 62.5% (n = 15) |  |  |
| Living with friends or sharing | 34.0% (n = 16) |  | 20.5% (n = 9) |  | 25.0% (n = 6) |  |  |
| Living alone | 10.6% (n = 5) |  | 25.0% (n = 11) |  | 12.5% (n = 3) |  |  |
| Current smoking | 83.3 % (n = 40) |  | 81.8 % (n = 36) |  | 84.2% (n = 16) |  |  |
| History of smoking | 23.1% (n = 9) |  | 22.5% (n = 9) |  | 31.8% (n = 7) |  |  |
| **AUDIT total score** | 5.8 (6.9) | 3.8-7.9 | 3.4 (5.1) | 1.8-5.0 | 3.2 (3.3) | 1.7-4.6 |  |
| **DASS total score** | 29.4 (15.1)^a***^ | 24.8-34.0 | 30.3 (12.0)^a***^ | 26.5-34.1 | 6.7 (7.2) | 3.6-9.8 |  |
| DASS stress score | 11.0 (5.4)^a***^ | 9.5-12.7 | 12.2 (4.6)^a***^ | 10.8-13.6 | 3.5 (2.9) | 2.2-4.7 |  |
| DASS anxiety score | 7.9 (4.8)^a***^ | 6.5-9.3 | 7.3 (4.8)^a***^ | 5.8-8.8 | 1.0 (2.4) | 0.0-2.1 |  |
| DASS depression score | 10.7 (6.3)^a***^ | 8.9-12.5 | 11.0 (5.1)^a***^ | 9.5-12.6 | 2.2 (3.3) | 0.8-3.5 |  |
| **Menstrual health** |  |  |  |  |  |  |  |
| Age of menarche [year] | 13.1 (2.0) | 12-5-13.7 | 13.5 (2.8) | 12.-14.3 | 12.3 (1.5) | 11.7-12.9 |  |
| History of amenorrhoea | 39.6%% (n = 19)^b*^ |  | 66.7% (n = 30)^a**^ |  | 16.7 % (n = 4) |  |  |
| Current amenorrhoea | 25.5% (n = 12) |  | 46.7% (n= 21)^a*^ |  | 16.7% (n = 4) |  |  |
| Duration of amenorrhoea [year] | 1.5 (1.6)^b***^ | 1.0-2.0 | 5.0 (3.0)^a***^ | 4.1-6.0 | 0.7 (1.4) | 0.0-1.3 |  |
| History of hormone contraception | 45.8% (n = 22) |  | 50.0% (n = 22) |  | 62.5 % (n = 15) |  |  |
| Current OCP | 83.3 % (n = 40) |  | 82.2 % (n = 37) |  | 83.3% (n = 20) |  |  |
| History of pregnancy | 8.2% (n = 4) |  | 17.8% (n = 8) |  | 17.4% (n = 4) |  |  |
| Expected reproductive lifespan [year] | 10.9 (6.1) ^a** b*^ | 9.1-12.8 | 15.5 (8.5) | 12.9-18.1 | 19.2 (11.8) | 14.2-24.2 |  |
| Reported reproductive lifespan [year] | 10.1 (6.2) ^a***^ | 8.2-11.9 | 13.0 (8.7) ^a*^ | 10.3-15.7 | 18.8 (12.0) | 13.7-23.8 |  |
| Reported/expected reproductive lifespan [% out total] | 85.4 (22.9) | 78.3-92.3 | 78.0 (29.0)^a*^ | 69.0-87.0 | 97.0 (2.0) | 96.0-98.0 |  |
| Amenorrhoea-adjusted/expected lifespan [% out total] | 82.4 (21.0)^a**^ | 76.1-88.7 | 63.3 (29.4)^a***^ | 55.2-77.3 | 96.5 (9.3) | 92.5-100.0 |  |
| **ED symptoms and behaviours** |  |  |  |  |  |  |  |
| EDE-Q Global score | 3.5 (1.0)^a***^ | 3.2-3.8 | 3.7 (1.3)^a***^ | 3.2-4.1 | 0.7 (1.0) | 0.3-1.2 |  |
| EDE-Q Restraint score | 3.4 (1.4)^a***^ | 3.0-3.8 | 3.7 (1.5)^a***^ | 3.3-4.2 | 1.0 (1.4) | 0.3-1.6 |  |
| EDE-Q Eating concern score | 3.1 (1.3)^a***^ | 2.7-3.4 | 3.1 (1.6)^a***^ | 2.6-3.6 | 0.1 (0.4) | -0.1-0.3 |  |
| EDE-Q Shape concern score | 4.0 (1.2)^a***^ | 3.6-4.3 | 4.1 (1.4)^a***^ | 3.7-4.6 | 1.0 (1.3) | 0.4-1.5 |  |
| EDE-Q Weight concern score | 3.6 (1.2)^a***^ | 3.3-3.9 | 3.5 (1.6)^a***^ | 3.0-4.0 | 0.8 (1.1) | 0.3-1.3 |  |
| Restricting behaviour | 71.4% (n = 35) ^b*^ |  | 44.4% (n = 20) |  | 8.3% (n = 2) |  |  |
| Monthly average/past 3 months | 6.8 (6.3)^a*** b*^ | 5.0-8.6 | 4.1 (6.2)^a*^ | 2.1-6.0 | 0.8 (3.3) |  |  |
| Binge eating episode | 64.6% (n = 31)^b***^ |  | 22.2% (n = 10) |  |  |  |  |
| Monthly average/past 3 months | 6.1 (9.3)^a*** b***^ | 3.4-8.9 | 4.1 (15.2) | -0.5-8.6 |  |  |  |
| Self-induced vomiting | 49.0% (n = 24)^b*^ |  | 26.7% (n = 12) |  |  |  |  |
| Monthly average/past 3 months | 6.2 (11.4) ^a*** b*^ | 2.9-9.5 | 6.4 (20.3) | 0.2-12.7 |  |  |  |
| Laxative/diuretics use | 30.6% (n = 15) |  | 24.4% (n = 11) |  | 4.2% (n = 1) |  |  |
| Monthly average/past 3 months | 2.2 (4.5) ^a*^ | 0.9-3.5 | 1.9 (4.9) | 0.4-3.4 | 0.0 (0.2) | 0.0-0.1 |  |
| Compensatory exercise | 79.6% (n = 39)^b**^ |  | 46.7% (n = 21) |  | 4.2% (n = 1) |  |  |
| Monthly average/past 3 months | 7.1 (6.1)^a***, b*^ | 5.3-8.8 | 3.9 (5.9)^a*^ | 2.1-5.7 | 0.4 (2.0) | -0.4-1.3 |  |
| **Physical activity** |  |  |  |  |  |  |  |
| Walking (hour/week) | 0.7 (0.7)^b*^ | 0.5-0.9 | 1.3 (1.3)^**a^ | 0.9-1.7 | 0.4 (0.6) | 0.2-0.7 |  |
| MPVA (hour/week) | 8.1 (8.3) | 5.6-10.5 | 13.0 (13.7) ^a**^ | 8.9-17.2 | 4.7 (5.9) | 2.2-7.2 |  |

*Notes:*  CI: confidence interval, n: sample size, *p: p-*value, SD: standard deviation, AN: anorexia nervosa, AUDIT: Alcohol Use Disorders Identification Test, BMI: body mass index, DASS: Depression, Anxiety and Stress Scale, EDE-Q: Eating Disorder Examination-Questionnaire, FE: first episode (≤ 3 years), HC: healthy controls, MPVA: moderate and vigorous physical activity, OCP: oral contraceptive pill, P: persistent (≥ 7 years).

ANOVA post-hoc Bonferroni and Chi squared tests, * *p* <0.05, ** *p* < 0.01, *** *p* < 0.001 versus a. HC and b. P-AN.

**Appendix 2.** Eating disorders (EDs) factors associations with total of fractures in first episode (FE-AN) and persistent anorexia nervosa (P-AN) groups.

| **Variable label** | FE-AN | | | | | P-AN | | | | |
| --- | --- | --- | --- | --- | --- | --- | --- | --- | --- | --- |
|  | **n** | **β** | **95% CI Lower** | **95% CI Upper** | ***p*** | **N** | **β** | **95% CI Lower** | **95% CI Upper** | ***P*** |
| Age [year] (log) | 47 | -0.011 | -1.092 | 1.015 | 0.941 | 45 | 0.501 | -0.257 | 1.762 | 0.140 |
| AN type | 48 | 0.248 | -0.283 | 3.594 | 0.092 | 42 | -0.102 | -3.461 | 1.839 | 0.539 |
| Duration of illness [month] (log) | 47 | 0.005 | -0.781 | 0.808 | 0.973 | 45 | 0.321 | -0.417 | 1.669 | 0.233 |
| Skin colour | 47 | -0.144 | -3.239 | 1.142 | 0.340 | 45 | -1.753 | -7.426 | 0.521 | 0.087 |
| Smoking history [yes] | 37 | -0.118 | -3.814 | 1.852 | 0.486 | 40 | -0.039 | -3.676 | 2.917 | 0.817 |
| Current BMI [kg/m2] | 41 | -0.001 | -0.374 | 0.371 | 0.994 | 44 | **-0.320** | **-1.237** | **-0.061** | **0.031*** |
| Lowest-ever BMI [kg/m^2^] | 40 | -0.024 | -0.501 | 0.435 | 0.887 | 44 | -0.235 | -1.302 | 0.177 | 0.132 |
| Highest-ever BMI [kg/m^2^] (log) | 40 | -0.038 | -1.970 | 1.560 | 0.815 | 42 | -0.042 | -2.179 | 1.676 | 0.793 |
| Δ BMI (Current - lowest ever BMI) [kg/m^2^] | 39 | 0.073 | -0.670 | 1.040 | 0.663 | 44 | -0.177 | -1.255 | 0.337 | 0.251 |
| Age of menarche [year] (log) | 45 | -0.108 | -2.457 | 1.187 | 0.486 | 43 | -0.088 | -1.976 | 1.102 | 0.569 |
| Current amenorrhoea [yes] | 45 | 0.060 | -1.940 | 2.866 | 0.699 | 45 | 0.177 | -0.930 | 3.674 | **0**.236 |
| History of amenorrhoea [yes] | 46 | 0.000 | -2.122 | 2.125 | 0.999 | 45 | 0.082 | -1.939 | 3.281 | 0.607 |
| Duration of amenorrhoea [year] (log) | 42 | 0.118 | -0.229 | 0.634 | 0.472 | **40** | **0.384** | **0.180** | **0.937** | **0.013*** |
| Current OCP [yes] | 46 | -0.047 | -3.055 | 2.246 | 0.760 | 45 | 0.113 | -1.910 | 4.200 | 0.454 |
| Reproductive lifespan [year] (log) | 41 | 0.221 | -0.139 | 0.381 | 0.352 | 41 | -0.011 | -0.181 | 0.171 | 0.956 |
| Loss of reproductive lifespan [%] (log) | 43 | 0.133 | -0.051 | 0.120 | 0.416 | 42 | 0.274 | -0.009 | 0.156 | 0.078 |
| History of hormone contraception [yes] | 46 | -0.144 | -2.955 | 1.052 | 0.344 | 44 | 0.049 | -2.301 | 3.059 | 0.777 |
| History of pregnancy [yes] | 47 | 0.048 | -3.396 | 4.521 | 0.776 | **45** | **-0.363** | **-7.029** | **-0.306** | **0.033*** |

*Notes:*  CI: confidence interval, n: sample size, *p: p*-value, AN: anorexia nervosa, BMI: body mass index, FE: first episode (≤ 3 years), OCP: oral contraceptive pill, P: persistent (≥ 7 years). Values in bold denote statistical significance in linear regressions (*p* < 0.05) after including age as covariates. *Statistically significant after including age, EDE-Q global score and AN subtype as covariates.

**Appendix 3.** Additional demographic, anthropometric, and clinical characteristics of female adults with first episode (FE) or persistent anorexia nervosa (P-AN), and of healthy controls.

| **Sample characteristics** | **FE-AN (n = 49)** | | | **P-AN (n = 45)** | | | **HC (n = 24)** | | |
| --- | --- | --- | --- | --- | --- | --- | --- | --- | --- |
|  | **Mean (SD) or frequencies (n)** | **95% CI** | **Mean (SD) or frequencies (n)** | | **95% CI** | **Mean (SD) or frequencies (n)** | | **95% CI** |  |
| Age [year] | 24.2 (5.9) ^b**^ | 22.6-25.9 | 28.3 (7.0) | | 26.2-30.4 | 27.4 (8.6) | | 23.3-31.6 |  |
| AN Restricting type | 36.7% (n = 18)^b***^ |  | 75% (n = 33) | |  |  | |  |  |
| AN Binge-eating/purging type | 63.3% (n = 31)^b***^ |  | 25% (n = 11) | |  |  | |  |  |
| Duration of illness [year] | 1.9 (0.8)^b***^ | 1.7-2.1 | 14.5 (6.6) | | 12.4-16.5 |  | |  |  |
| AN onset age [year] | 21.7 (5.9)^b***^ | 19.9-23.4 | 16.1 (4.6) | | 14.5-17.8 |  | |  |  |
| **BMI [kg/m^2^]** |  |  |  | |  |  | |  |  |
| Current BMI [kg/m^2^] | 17.9 (2.5)^a*** b**^ | 17.1-18.7 | 15.8 (1.9)^a***^ | | 15.2-16.4 | 22.6 (3.7) | | 20.8-24.4 |  |
| Lowest-ever BMI [kg/m^2^] | 16.1 (2.3)^a*** b***^ | 15.3-16.8 | 13.9 (1.7)^a***^ | | 13.4-14.4 | 20.5 (2.9) | | 19.1-21.9 |  |
| Highest-ever BMI [kg/m^2^] | 21.4 (3.4)^a** b*^ | 20.4-22.5 | 19.6 (2.6)^a***^ | | 18.8-20.4 | 24.0 (4.2) | | 22.0-26.0 |  |
| **Skin colour** |  |  |  | |  |  | |  |  |
| Fair or very fair | 59.2% (n = 29) |  | 77.5% (n = 35)^a*^ | |  | 47.4% (n = 9) | |  |  |
| Olive | 14.3% (n = 7) |  | 11.4% (n = 5) | |  | 31.6% (n = 6) | |  |  |
| Brown or dark | 26.5% (n = 13) |  | 9.1% (n = 4) | |  | 21.1% (n = 4) | |  |  |
| **Occupation** |  |  |  | |  |  | |  |  |
| Working | 44.7% (n = 21) |  | 50.0% (n = 21) | |  | 42.1% (n = 8) | |  |  |
| Unemployed or on unpaid leave | 12.8% (n = 6) |  | 26.2% (n = 11) | |  | 15.8% (n = 3) | |  |  |
| Student | 42.6% (n = 20) |  | 23.8% (n = 10) | |  | 42.1% (n = 8) | |  |  |
| **Household** |  |  |  | |  |  | |  |  |
| Living with family | 55.3% (n = 26) |  | 55.8% (n = 24) | |  | 68.4% (n = 13) | |  |  |
| Living with friends or sharing | 34.0% (n = 16) |  | 20.9% (n = 9) | |  | 26.3% (n = 5) | |  |  |
| Living alone | 10.6% (n = 5) |  | 23.3% (n = 10) | |  | 5.3% (n = 1) | |  |  |
| **Current smoking** | 2.6% (n = 1) |  | 5.1% (n = 2) | |  | 0.0% (n = 0) | |  |  |
| **History of smoking** | 23.1% (n = 9) |  | 20.5% (n = 8) | |  | 27.8% (n = 5) | |  |  |
| **AUDIT total score** | 5.8 (6.9) | 3.8-7.9 | 3.5 (5.2) | | 1.8-5.1 | 2.4 (2.7) | | 1.1-3.7 |  |
| **DASS total score** | 29.4 (15.1)^a***^ | 24.8-34.0 | 30.2 (12.1)^a***^ | | 26.3-34.1 | 7.7 (7.8) | | 3.9-11.6 |  |
| DASS stress score | 11.0 (5.4)^a***^ | 9.5-12.7 | 12.1 (4.6)^a***^ | | 10.8-13.6 | 3.7 (3.0) | | 20.2-6.0 |  |
| DASS anxiety score | 7.9 (4.8)^a***^ | 6.5-9.3 | 72.3 (4.8)^a***^ | | 5.8-8.8 | 1.3 (2.7) | |  |  |
| DASS depression score | 10.7 (6.3)^a***^ | 8.9-12.5 | 11.0 (5.1)^a***^ | | 9.5-12.6 | 2.6 (3.5) | | 1.0-7.0 |  |
| **Menstrual health** |  |  |  | |  |  | |  |  |
| Age of menarche [year] | 13.1 (2.0) | 12-5-13.7 | 13.5 (2.8) ^a*^ | | 12.7-14.34 | 11.9 (1.4) | | 11.3-12.6 |  |
| History of amenorrhoea | 39.6% (n = 19)^b*^ |  | 78.2% (n = 30)^a**^ | |  | 21.1 % (n = 4) | |  |  |
| Current amenorrhoea | 25.5% (n = 12)^b*^ |  | 45.5% (n= 20)^a*^ | |  | 0.0% (n = 40) | |  |  |
| Duration of amenorrhoea [year] | 1.5 (1.6)^b***^ | 1.0-2.0 | 5.1 (2.9)^a***^ | | 4.2-6.1 | 0.7 (1.54) | | 0.2-1.6 |  |
| History of hormone contraception | 45.8% (n = 22) |  | 48.8% (n = 21) | |  | 57.9% (n = 11) | |  |  |
| Current OCP | 83.3 % (n = 40) |  | 81.8 % (n = 36) | |  | 84.2% (n = 16) | |  |  |
| Duration of hormone contraception [year] | 0.5 (1.1) ^a***^ | 0.1-0.8 | 1.3 (2.9) ^a**^ | | 0.4-2.1 | 4.2 (7.2) | | 1.2-7.3 |  |
| History of pregnancy | 8.2% (n = 4) |  | 15.9% (n = 7) | |  | 10.5% (n = 2) | |  |  |
| Expected reproductive lifespan [year] | 10.9 (6.1)^b*^ | 9.1-12.8 | 14.9 (7.7) | | 12.5-17.3 | 15.4 (8.7) | | 11.9-14.8 |  |
| Reported reproductive lifespan [year] | 10.1 (6.2) | 8.2-11.9 | 12.4 (7.7) | | 9.9-14.8 | 14.9 (8.8) | | 10.7-19.2 |  |
| Reported/expected reproductive lifespan [% out total] | 85.4 (22.9) | 78.3-92.3 | 77.3 (29.2)^a*^ | | 68.1-85.5 | 95.9 (1.9) | | 95.0-96.9 |  |
| Amenorrhoea-adjusted/expected lifespan [% out total] | 82.4 (21.0)^a*** b***^ | 76.1-88.7 | 63.4 (29.3)^a***^ | | 54.3-72.6 | 95.9 (10.4) | | 90.9-100.0 |  |
| **ED symptoms and behaviours** |  |  |  | |  |  | |  |  |
| EDE-Q Global score | 3.5 (1.0)^a***^ | 3.2-3.8 | 3.7 (1.4)^a***^ | | 3.2-4.1 | 0.8 (1.1) | | 0.2-1.3 |  |
| EDE-Q Restraint score | 3.4 (1.4)^a***^ | 3.0-3.8 | 3.8 (1.4) ^a***^ | | 3.6-4.6 | 0.9 (1.5) | | 0.0-1.2 |  |
| EDE-Q Eating concern score | 3.1 (1.3)^a***^ | 2.7-3.4 | 3.1 (1.6) ^a***^ | | 2.4-3.8 | 0.2 (0.5) | |  |  |
| EDE-Q Shape concern score | 4.0 (1.2)^a***^ | 3.6-4.3 | 4.1 (1.4) ^a***^ | | 3.6-4.9 | 0.9 (1.4) | | 0.2 -0.8 |  |
| EDE-Q Weight concern score | 3.6 (1.2)^a***^ | 3.3-3.9 | 3.5 (1.6) ^a***^ | | 3.6-4.4 | 0.8 (1.2) | | 0.0-0.8 |  |
| Restricting behaviour | 71.4% (n = 35) ^a*** b*^ |  | 27.3% (n = 12) | |  |  | |  |  |
| Monthly average/past 3 months | 6.8 (6.3)^a*** b*^ | 5.0-8.6 | 6.64 (20.5) | | 0.2-13.0 |  | |  |  |
| Binge eating episode | 64.6% (n = 31)^b***^ |  | 22.7% (n = 10) | |  |  | |  |  |
| Monthly average/past 3 months | 6.1 (9.3)^a*** b***^ | 3.4-8.9 | 1.9 (4.9) | | 0.4-3.45 |  | |  |  |
| Self-induced vomiting | 49.0% (n = 24)^b*^ |  | 47.7% (n = 21) | |  |  | |  |  |
| Monthly average/past 3 months | 6.2 (11.4) ^a*** b*^ | 2.9-9.5 | 4.0 (5.9)^a*^ | | 2.2-5.9 |  | |  |  |
| Laxative/diuretics use | 30.6% (n = 15) |  | 43.2% (n = 19) ^a*^ | |  | 10.5% (n = 2) | |  |  |
| Monthly average/past 3 months | 2.2 (4.5) ^a*^ | 0.9-3.5 | 4.0 (6.3)^a*^ | | 2.1-6.0 | 0.9 (3.7) | |  |  |
| Compensatory exercise | 79.6% (n = 39)^b**^ |  | 20.5% (n = 9) | |  |  | |  |  |
| Monthly average/past 3 months | 7.1 (6.1) ^b*^ | 5.3-8.8 | 4.0 (15.4) | | -0.7-8.7 |  | |  |  |
| **Physical activity** |  |  |  | |  |  | |  |  |
| Walking (hour/week) | 0.7 (0.7)^b*^ | 0.5-0.9 | 1.3 (1.4) ^a**^ | | 0.9-1.7 | 0.2 (0.3) | | 0.1-0.4 |  |
| MPVA (hour/week) | 8.1 (8.3) | 5.6-10.5 | 13.2 (13.9) ^a**^ | | 9.0-17.4 | 3.2 (2.3) | | 2.1-4.4 |  |

*Notes:*  CI: confidence interval, n: sample size, *p: p-*value, SD: standard deviation, AN: anorexia nervosa, AUDIT: Alcohol Use Disorders Identification Test, BMI: body mass index, DASS: Depression, Anxiety and Stress Scale, EDE-Q: Eating Disorder Examination-Questionnaire, FE: first episode (≤ 3 years), HC: healthy controls, MPVA: moderate and vigorous physical activity, OCP: oral contraceptive pill, P: persistent (≥ 7 years).

ANOVA post-hoc Bonferroni and Chi squared tests, * *p* <0.05, ** *p* < 0.01, *** *p* < 0.001 versus a. HC and b. P-AN.

**Apprendix 4.** Fractures in female adults with first episode (FE-AN) or persistent (P-AN) anorexia nervosa (AN), and in healthy controls.

| **Reported fractures** | **FE-AN (n = 49)** | **P-AN (n = 44)** | **HC (n = 19)** |
| --- | --- | --- | --- |
|  | **Mean [Min-Max] or frequencies (n)** | **Mean [Min-Max] or frequencies (n)** | **Mean [Min-Max] or frequencies (n)** |
| **All fractures, including stress fractures** |  |  |  |
| Lifetime history | 33.3% (n = 16) ^a**^ | 38.6% (n = 17) ^a**^ | 5.3% (n =1) |
| Lifetime number | 1.3 [0-20] | 1.9 [0-19]^a*^ | 0.1 [0-2] |
| Before AN onset history | 26.5% (n=13) | 25.0 (n = 11) |  |
| Before AN onset number | 2.2 [0-7] | 0.5 [0 -2] |  |
| After AN onset history | 12.2% (n = 6) | 22.7% (n = 10) |  |
| After AN onset number | 2.0 [ 1 – 3] ^b***^ | 7.0 [6 – 8] |  |
| **Fractures** |  |  |  |
| Lifetime history | 29.2% (n = 14)^a*^ | 38.6% (n = 17)^a**^ | 5.3% (n =1) |
| Lifetime number | 1.0 [0-17] ^a*^ | 1.7 [0-18]^a*^ | 0.1 [0-2] |
| Before AN onset history | 22.4% (n = 11) | 25.0% (n = 11) |  |
| Before AN onset number | 0.8 [0-17] | 0.4 [0-4] |  |
| After AN onset history | 10.2% (n = 5) | 22.7% (n = 10) |  |
| After AN onset number | 0.2 [0-2] ^b*^ | 0.6 [0-5] |  |
| **Stress fractures** |  |  |  |
| Lifetime history | 20.8% (n = 10)^a**b*^ | 9.1% (n = 4)^a**^ | 0% (n = 0) |
| Lifetime number | 0.3 [0-3] ^a*^ | 0.2 [0-3] |  |
| Before AN onset history | 12.2% (n = 6) | 2.3% (n = 1) |  |
| Before AN onset number | 0.8 [0-3]^b**^ | 0.1 [0-1] |  |
| After AN onset history | 6.8% (n = 3) | 6.7% (n=3) |  |
| After AN onset number | 0.4 [0-1] ^b**^ | 2.0 [1-3] |  |
| Fracture history^1^ |  |  | *Reference* |
| Wald | 4.1 | 5.1 | 5.1 |
| Odds ratio | 8.9 | 11.3 |  |
| 95% CI | 1.1-74.0 | 1.4-93.0 |  |
| *p* | **0.042** | **0.024** | 0.077 |

*Notes:*  CI: confidence interval, n: sample size, *p: p*-value, AN: anorexia nervosa, FE: first episode (≤ 3 years), HC: healthy controls, P: persistent (≥ 7 years).

Comparisons between groups by Independent T-Test and Chi-squared tests after Fisher’s Exact Test, * *p* <0.05, ** *p* < 0.01, ****p* < 0.001 versus a. HC and b. P-AN

^1^Logistic regression for fracture history in FE-AN and P-AN, and healthy controls groups, adjusted for age. Values in bold denote statistical significance (*p* < 0.05).

**Appendix 5.** Eating disorders (EDs) factors associations with total of fractures in first episode (FE-AN) and persistent anorexia nervosa (P-AN) groups.

| **Variable label** | FE-AN | | | | | P-AN | | | | |
| --- | --- | --- | --- | --- | --- | --- | --- | --- | --- | --- |
|  | **n** | **β** | **95% CI Lower** | **95% CI Upper** | ***p*** | **N** | **β** | **95% CI Lower** | **95% CI Upper** | ***P*** |
| Age [year] (log) | 47 | -0.011 | -1.092 | 1.015 | 0.941 | **44** | **0.309** | **0.0.051** | **2.305** | **0.041*** |
| AN type | 48 | 0.248 | -0.283 | 3.594 | 0.092 | 44 | 0.118 | -1.601 | 3.722 | 0.426 |
| Duration of illness [month] (log) | 47 | 0.005 | -0.781 | 0.808 | 0.973 | 44 | 0.327 | -0.316 | 1.730 | 0.170 |
| Skin colour | 48 | -0.140 | -3.182 | 1.148 | 0.349 | 44 | -1.804 | -7.297 | 0.412 | 0.079 |
| Smoking history [yes] | 38 | -0.105 | -3.660 | 1.923 | 0.532 | 39 | -0.003 | -3.200 | 3.265 | 0.984 |
| Current BMI [kg/m2] | 40 | -0.020 | -0.477 | 0.422 | 0.901 | 42 | -0.258 | -1.145 | 0.078 | 0.086 |
| Lowest-ever BMI [kg/m^2^] | 39 | -0.085 | -0.628 | 0.373 | 0.608 | 42 | -0.193 | -1.199 | 0.275 | 0.213 |
| Highest-ever BMI [kg/m^2^] (log) | 40 | -0.038 | -1.970 | 1.560 | 0.815 | 41 | 0.020 | -1.875 | 2.124 | 0.901 |
| Δ BMI (Current - lowest ever BMI) [kg/m^2^] | 39 | 0.081 | -0.635 | 1.040 | 0.627 | 42 | -1.125 | 0.741 | 0.365 | 0.412 |
| Age of menarche [year] (log) | 45 | -0.108 | -2.457 | 1.187 | 0.486 | 42 | -0.126 | -2.148 | 0.881 | 0.402 |
| Current amenorrhoea [yes] | 46 | 0.052 | -1.910 | 2.685 | 0.735 | 44 | 0.240 | -0.370 | 4.132 | **0**.099 |
| History of amenorrhoea [yes] | 47 | 0.007 | -2.034 | 2.127 | 0.964 | 44 | 0.073 | -1.929 | 3.152 | 0.630 |
| Duration of amenorrhoea [year] (log) | 42 | 0.118 | -0.229 | 0.634 | 0.472 | **39** | **0.327** | **0.027** | **0.904** | **0.038*** |
| Current OCP [yes] | 47 | -0.049 | -3.048 | 2.192 | 0.743 | 44 | 0.068 | -2.343 | 3.718 | 0.649 |
| Reproductive lifespan log | 41 | 0.221 | -0.139 | 0.381 | 0.352 | 41 | -0.049 | -0.195 | 0.152 | 0.804 |
| Loss of reproductive lifespan log | 43 | 0.133 | -0.051 | 0.120 | 0.416 | 42 | 0.238 | -0.016 | 0.147 | 0.114 |
| History of hormone contraception [yes] | 46 | -0.148 | -2.935 | 0.998 | 0.326 | 43 | -0.003 | -2.672 | 2.628 | 0.987 |
| History of pregnancy [yes] | 47 | 0.047 | -3.372 | 4.469 | 0.779 | **44** | **-0.336** | **-6.850** | **-0.321** | **0.032*** |

*Notes:*  CI: confidence interval, n: sample size, *p: p*-value, AN: anorexia nervosa, BMI: body mass index, FE: first episode (≤ 3 years), MPVA: moderate and vigorous physical activity, OCP: oral contraceptive pill, P: persistent (≥ 7 years). Values in bold denote statistical significance in linear regressions (*p* < 0.05) after including age as covariates. *Statistically significant after including age, EDE-Q global score and AN subtype as covariates.
